# Improved attenuation correction registration for FDG PET/CT images using data-driven gating (DDG)-based motion match

**DOI:** 10.1186/s40658-026-00896-y

**Published:** 2026-05-24

**Authors:** Zoë Wilson, Manjeet Kuhar, Kuan-Hao Su, Robert Johnsen, Kevin M. Bradley, Daniel R. McGowan

**Affiliations:** 1 Department of Medical Physics and Clinical Engineering, Oxford University Hospitals Foundation Trust, Oxford, UK; 2 GE HealthCare, Waukesha, WI USA; 3 Wales Research and Diagnostic PET Imaging Centre, Cardiff University, Cardiff, Wales; 4 Department of Oncology, Oxford University, Oxford, UK

**Keywords:** PET, CT, Attenuation correction, Respiratory motion

## Abstract

**Background:**

Advancements in PET technologies and reconstruction methods have improved spatial resolution and noise in PET/CT, making respiratory motion artefacts more impactful on image quality. The motion-match CT (MMCT) algorithm estimates the phase of a helical CT and warps it towards the end expiration of a patient’s respiratory cycle. This MMCT-attenuation correction (MMCTAC) can then be used to reconstruct whole body FDG PET/CT data. The performance of these images were compared to those reconstructed using the uncorrected clinical standard attenuation correction from helical CT (normAC), indicating the proportion of cases where MMCTAC images could reduce motion artefact and improve lesion detectability.

**Methods:**

A sequential cohort of 145 whole-body FDG PET/CT scans was previously evaluated for a data-driven gating study, which identified the gated region of interest (ROI) in 98 high-motion patients. 23 of these high-motion patients had small lesions (n=36) identified in the ROI by a clinician to undergo qualitative analysis comparing the two attenuation correction maps used. PET data were reconstructed using BSREM iterative reconstruction with 3 different portions of the collected data: 1. Ungated 6 min, 2. Ungated 3 min, 3. Quiescent period gated. These reconstructions were generated using normAC or MMCTAC, totaling six reconstructions for semi-quantitative analysis and clinician evaluation. An experienced radiologist ranked the 6 images and scored them on a 5-point Likert Scale for lesion detectability, diagnostic confidence, and image quality.

**Results:**

In 52% (n=12) of patients with lesions, the clinician ranked all three MMCTAC images higher than the three normAC equivalent reconstructions. Lesion detectability and diagnostic confidence scores retained positive mean differences across the three MMCTAC reconstructions compared to normAC. Similarly, the SUVmax values for 36 lesions demonstrated a significant increase (*p* < 0.05) for MMCTAC versus normAC images across all three reconstructions.

**Conclusions:**

The MMCT algorithm successfully enables the phase-matching of a helical CT to the PET data to reduce the presence of respiratory motion artefact for FDG PET/CT images. Overall, the motion-matched CTAC image scores demonstrated the algorithm’s efficacy at reducing the motion artefacts present in FDG PET/CT images, improving diagnostic capability for assessment of lesions in the thorax and upper abdomen.

## Background

Respiratory motion remains a prevalent source of artefact in PET/CT and an area of PET imaging research that continues to push boundaries in the field. The clinical impact of these artefacts is well characterized [1, 2] with PET-focused respiratory gated reconstruction methods becoming mainstay protocols within hospitals [3–5]. As detector technology, time-of-flight resolution and Bayesian penalized reconstruction methods continue to sharpen lesion contrast and reduce statistical noise, even sub-centimetre misregistration between PET and CT becomes visually obvious and can affect quantitative accuracy, staging, and therapy response assessment [6–8].

The successfully fused PET/CT image provides an essential combination of the structural information from the CT and functional PET data. The incorporation of CT data with PET both allows for improved spatial localization of radiopharmaceutical uptake and the generation of an attenuation correction map converted from Hounsfield units, and scaled up to 511 keV. This map can be used during PET image reconstruction known as CT attenuation correction (CTAC).

A motion-matched CT (MMCT) algorithm has been developed to produce a phase-matched MMCT-attenuation correction (MMCTAC) map to be used in the PET reconstruction. MMCT aims to estimate the phase of a helical CT and warp it towards the end expiration of a patient’s respiratory cycle. The success of this algorithm has been shown in a series of patients which had 4DCT FMISO scans to act as a ground truth for comparison with the MMCT, significantly improving attenuation correction alignment between the fused modalities without requiring external motion tracker devices or extending acquisition protocol times [9]. A comprehensive explanation of the MMCT algorithm pipeline can be found in this previous study.

This study aims to build upon this work in extending the application of the MMCT algorithm to assess its performance with F18-FDG full body scans, while previous studies have only applied MMCT to a single bed position. This will also provide the first report of blinded clinician scoring for diagnostic confidence and image preference of PET images reconstructed with MMCTACs, compared to using the attenuation correction maps from the current clinical standard helical CT.

## Materials and methods

### Patient data

This study analysed a sequential cohort of 145 whole-body F18-FDG scans collected from August 2018 using GE’s Discovery 710 or Discovery 690 scanners.

Inclusion criteria for patients in this study were degree of motion and presence of at least one lesion in the lungs or liver, which are organs of interest most effected by respiratory motion [10]. The degree of respiratory-like motion present during a given bed position during an acquisition is quantified by the R value. This is identified in GE HealthCare’s DDG algorithms by extracting the signal-to-noise ratio of respiration-like frequencies [11, 12]. A threshold of R>15 is used in GE’s commercially available DDG algorithms to indicate a degree of respiratory-like motion sufficient to warrant motion correction gating and this threshold was applied in this study

The PET images were previously assessed for a data-driven gating study, identifying the gated region of interest (ROI) most affected by respiratory motion (encompassing the liver and lower lungs) in the 98 patients with high-motion (R>15) [13]. 23 of these high motion patients had lesions (n=36) identified in the ROI by a clinician to undergo qualitative analysis. Only lesions *łe *1 cm were included for analysis. This lesion threshold was set by an experienced clinician based on practical experience with lesion detectability thresholds in FDG PET scans. Larger lesions are less sensitive to respiratory motion [14].

For each patient, a whole-body helical CT was performed for PET attenuation correction using 100–120 kVp, 150-200 mAs. Injected activity was 4 MBq/kg and an FDG uptake time of 90 min.

#### Reconstructions

PET data were reconstructed using GE HealthCare’s BSREM iterative reconstruction algorithm (Q.Clear, *β * = 400 [15]) with 3 different portions of the collected data: 1. Static 6 min, 2. Static 3 min, 3. Quiescent period gated. These reconstructions were generated using attenuation correction maps generated from the clinical standard helical CT (normAC) or using the MMCTAC, providing a total of 6 reconstructions for semi-quantitative analysis and clinician assessment. Each image was reconstructed with Q.Clear, following the local clinical standard. The Static reconstructions are ungated.

Reconstruction 3 uses GE’s DDG algorithm commercially known as Q.Static, utilizing a phase-binning method that retains only 50% of the counts in each breathing cycle with the least motion [16]. Q.Static is applied to six minutes of data, resulting in the Q.Static image reconstruction using an equivalent number of counts to the three-minute reconstruction. Hence the inclusion of Reconstruction 1 to compare the relative utility of an ungated equal length acquisition. Reconstruction 2 just uses the first three minutes of acquired data per bed position. This is included to compare images reconstructed using the same number of counts, such that the effects of the respiratory motion correction methods can be fairly assessed across the reconstruction and attenuation correction methods.

### Patient data analysis

An experienced radiologist scored the 6 blinded PET/CT reconstructions for all 23 studies on a 5-point Likert Scale for lesion detectability, diagnostic confidence, and image quality [17]. A score of 1 is not diagnostic, 2 is poor, 3 is satisfactory, 4 is good, and 5 is excellent. The radiologist also identified 36 lesions and recorded the maximum Standard Uptake Value (SUVmax) for these lesions across all 6 reconstructions. The radiologist was presented with the 6 blinded and randomized reconstructions for each patient separately to enable a comparison between the different images. The Shapiro-Wilk test indicated a non-normal distribution for results therefore a Wilcoxon signed-rank test was used to identify significant differences across these values between reconstructions.

An additional qualitative score for motion artefact was included to indicate the presence of motion artefact identified by the clinician from a scale of 0-3 (0 = minimal, 1 = visible, 2 = moderate, 3 = marked).

The clinician also provided an overall rank of image preference across the different reconstructions.

## Results

### Image examples

**Fig. 1:**
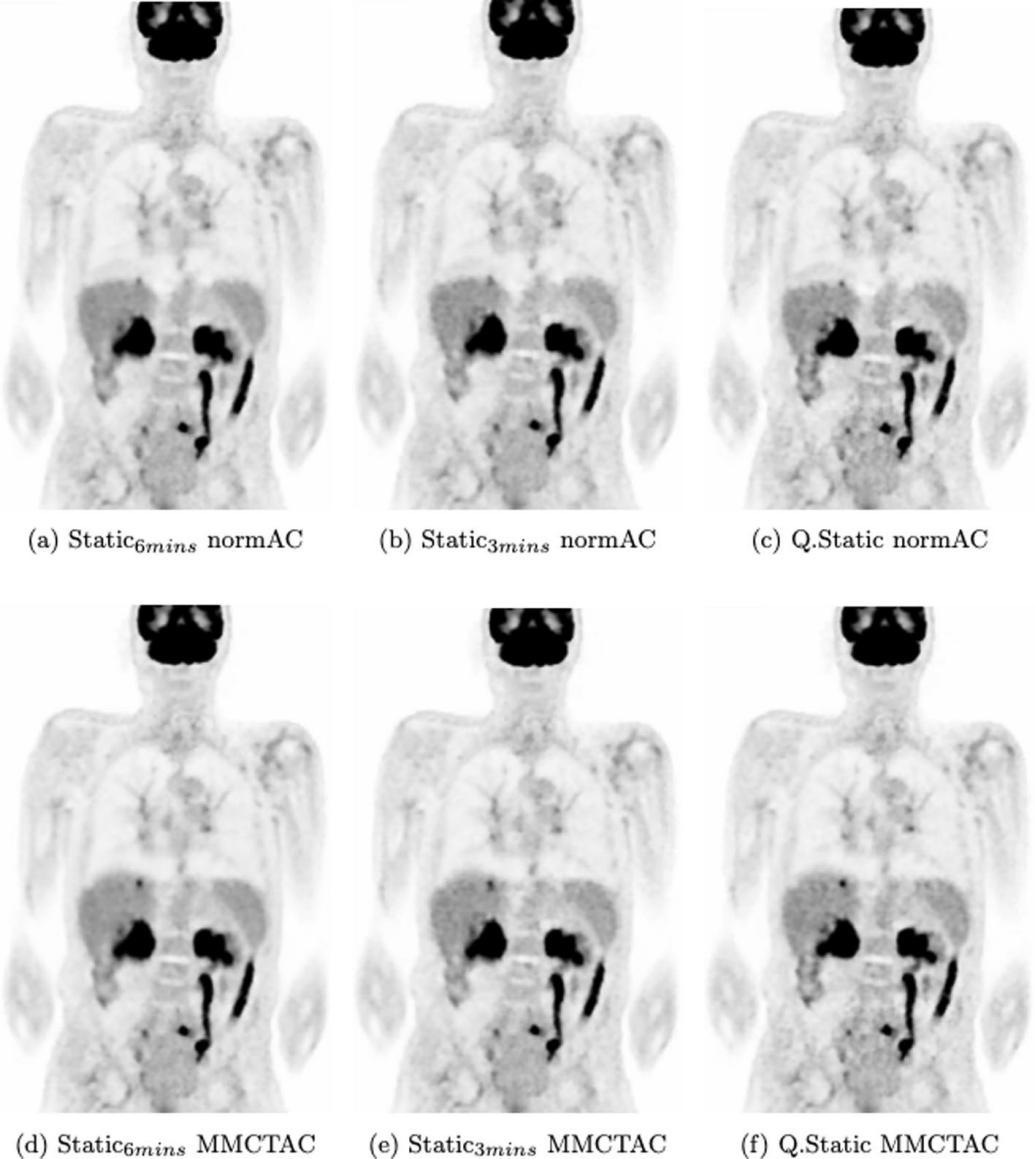
Comparison of PET/CT reconstructions using normAC **a**–**c** and MMCTAC **d**–**f**, taken from the same coronal image slice. The effect of the MMCTAC improving the localization of the lesion within the liver is clearly visible. Static 6 min reconstructions exhibited normAC lesion SUVmax = 3.2, MMCTAC lesion SUVmax = 5.3 (on SUV scale 0-8). These images are reconstructed from a patient included in this study, scanned at Oxford University Hospitals

A full series of all six reconstructions performed on a patient included in this study are depicted in Fig. 1 to demonstrate the effect of using normAC versus MMCTAC to reconstruct images with high motion, as well as to demonstrate the different reconstruction types included for analysis in this study.

### SUVmax

**Fig. 2:**
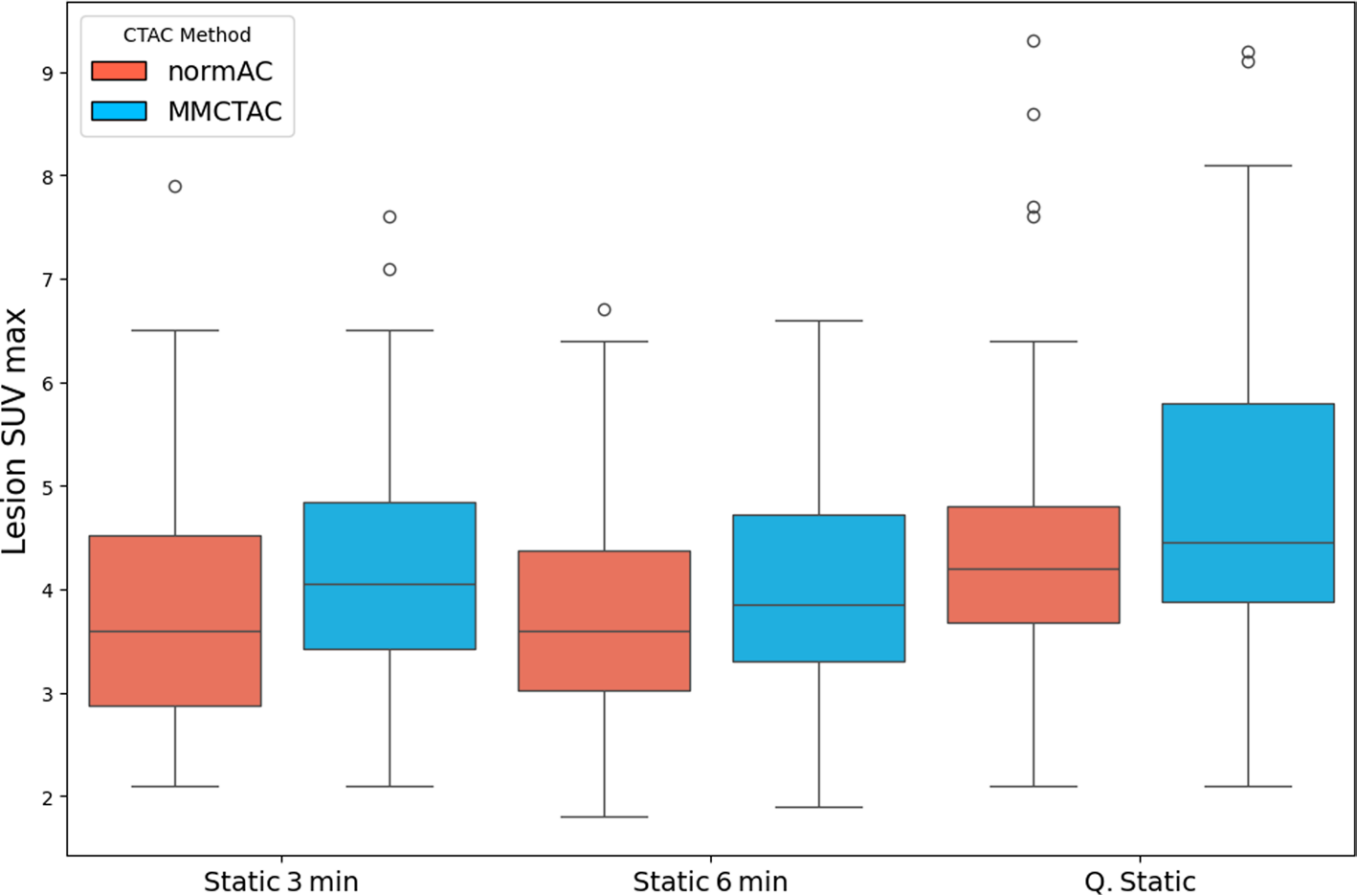
Box plots depicting the spread of SUVmax values for the 36 lesions included in this study, across the 6 reconstructions. Orange boxes indicate normAC, blue boxes indicate MMCTAC

The SUVmax values for the 36 lesions identified are summarized in the box plot in Fig. 2.

**Table 1:** Summary table with the Wilcoxon signed-rank test statistics and p-values comparing the SUVmax values between the normAC and MMCTAC methods for each of the three reconstruction types

Reconstruction	Test statistic	*p*-Value
Static$_{6\text {min}}$	137.0	0.017
Static$_{3\text {min}}$	77.5	0.002
Q.Static	43.6	0.001

The SUVmax values compared across the 6 reconstructed images for 36 lesions demonstrated a significant increase (*p* < 0.05) for MMCTAC versus normAC images across all three reconstructions, shown in Table 1.

When comparing across the reconstruction types, Q.Static reconstructions had significantly higher (*p* < 0.05) SUVmax values compared to ungated reconstructions for both normAC and MMCTAC, as expected from previous studies[5]. However, it can be seen in Table 2 the improvement in lesion SUVmax for the full counts Static 6 min reconstruction compared to the Static 3 min is only significant (*p* = 0.02) when MMCT is applied.

**Table 2:** Summary table with the Wilcoxon signed-rank test *p*-values comparing the SUVmax values between the three reconstruction types for normAC and MMCTAC methods

Recon comparison	CTAC	*p*-Value
Static$_{6\text {min}}$ and Static$_{3\text {min}}$	NormAC	0.051
	MMCTAC	0.02
Q.Static and Static$_{6\text {min}}$	NormAC	< 0.001
	MMCTAC	< 0.001
Q.Static and Static$_{3\text {min}}$	NormAC	< 0.001
	MMCTAC	< 0.001

#### Clinician scores

**Table 3:** Mean ± standard error of clinician scores for lesion detectability, diagnostic confidence, and image quality across the six reconstructions

Recon	CTAC	Lesion Det.	Diagnostic Conf.	Image Qual.	Motion Art.
Static$_{6\text {min}}$	NormAC	3.41 ± 0.14	3.45 ± 0.14	**3.52 ± 0.11**	1.67 ± 0.20
	MMCTAC	**3.55 ± 0.15**	**3.55 ± 0.16**	3.48 ± 0.12	**1.19 ± 0.24**
Static$_{3\text {min}}$	NormAC	2.82 ± 0.17	2.64 ± 0.14	2.48 ± 0.11	1.67 ± 0.20
	MMCTAC	**3.09 ± 0.16**	**2.82 ± 0.13**	**2.52 ± 0.11**	**1.19 ± 0.24**
Q.Static	NormAC	3.50 ± 0.14	3.23 ± 0.16	2.61 ± 0.12	1.67 ± 0.20
	MMCTAC	**3.55 ± 0.14**	**3.32 ± 0.15**	2.61 ± 0.12	**1.19 ± 0.24**

Scores range from 1 (not diagnostic) to 5 (excellent). Motion artefact scores are included, ranging from 0 (minimal) to 3 (marked). Best values for each metric are bolded

The clinician’s mean scores for the three qualitative metrics are summarized in Table 3. A positive mean difference for MMCTAC images compared to normAC reconstructions are observed across all three reconstructions for Lesion Detectability and Diagnostic Confidence. A summary table of the *p*-values and Wilcoxon signed-rank test statistics are in Table 4.

The only significant increase in Diagnostic Confidence was observed for the Static 3minute reconstructions, with the MMCTAC scores significantly higher (2.82 ± 0.13) than the normAC reconstructions (2.64 ± 0.14). The same result was seen in the Lesion Detectability scores with no significant difference across the normAC and MMCTAC reconstructions for Static 6 min or Q.Static gated image. Lesion Detectability was significantly higher in MMTAC Static 3 min reconstructions (3.09 ± 0.16) compared to normAC reconstructed images (2.82 ± 0.13). Image Quality yielded no statistically significant difference for clinician scoring between MMCTAC and normAC methods across the 3 reconstructions, with the Q.Static gated images being scored identically for both CTAC methods.

**Table 4:** Summary table with the Wilcoxon signed-rank test *p*-values comparing the three qualitative metric clinician scores between the normAC and MMCTAC methods for each of the three reconstruction types

Reconstruction	Lesion Det.	Diagnostic Conf.	Image quality
Static$_{6\text {min}}$	0.18	0.48	0.65
Static$_{3\text {min}}$	0.03	0.03	0.32
Q.Static	0.32	0.16	N/A

**Table 5:** Wilcoxon signed-rank test *p*-values comparing reconstruction types’ clinician scores for lesion detectability, diagnostic confidence, and image quality

Recon	CTAC	Lesion Det.	Diagnostic Conf.	Image Qual.
Static$_{6\text {min}}$ and Static$_{3\text {min}}$	NormAC	<0.001	<0.001	<0.001
	MMCTAC	<0.001	<0.001	<0.001
Q.Static and Static$_{6\text {min}}$	NormAC	0.3	0.3	<0.001
	MMCTAC	0.26	0.26	<0.001
Q.Static and Static$_{3\text {min}}$	NormAC	0.003	0.003	0.08
	MMCTAC	0.01	0.01	0.32

The comparison of reconstruction types within each AC method (summarized in Table 5), the lesion detectability and diagnostic confidence between Static 6 min and Q.Static reconstructions are not significantly different (p>0.05) for both normAC and MMCTAC. Image quality for Static 6 min reconstructions is significantly higher than both the half counts reconstructions, as would be expected with reduced noise due to greater counts for the longer acquisition time. No changes in significance level for the pairwise comparisons were seen between normAC and MMCTAC images.

The mean motion artefact scores demonstrated the efficacy of the MMCTAC method (1.19 ± 0.20) over normAC (1.67 ± 0.24), with Wilcoxon testing yielding a significant decrease in motion artefact score (p=0.015) across all 23 patients. The radiologist gave the same motion artefact score to each reconstruction for a given patient and AC method so no difference in artefact scores could be compared across the reconstruction types. Similarly, in 52% (n=12) of the patients with lesions, the clinician ranked all three MMCTAC images higher than the three normAC equivalent reconstructions.

## Discussion

The ability to apply the MotionMatch CT algorithm retrospectively to the acquired helical CT, with no additional hardware and without the additional dose of a 4DCT allows the acquisition workflow to remain identical for the ease of technologists and patient experience.

The results of this study are in accordance with the previous MMCT FMISO cohort, with reduced respiratory motion artefacts present across the MMCTAC reconstructions, compared to those using the clinical standard helical CT [9]. No instance of algorithm failure or the introduction of additional artefacts in these regions of interest were present in this cohort. This indicates a clinically feasible method to reduce CTAC mismatch due to respiratory motion for high motion patients with lesions in the thorax and liver. Although the statistical testing of Likert Scores from the clinician assessing the three qualitative metrics did not demonstrate a statistically significant difference in the MMCTAC images compared to normAC, they maintained a positive mean difference for MMCTAC scores across all three reconstruction types, with a single exception being the Static 6 min Image Quality scores.

Overall, clinician rankings indicate a preference for motion-corrected PET/CT images; this was further validated by a statistically significant decrease (p = 0.015) invisible motion artefact score. In 52% of cases, all three MMCTAC reconstruction types were ranked higher than the normAC equivalent reconstructions. Other patients had one or more of the MMCTAC reconstructions ranked equivalent to normAC. No normAC images were ranked higher than the MMCTAC equivalent reconstruction. MMCT had not previously been applied to ungated images and this study demonstrates it can effectively reduce artefacts without being applied in tandem with DDG. This artefact reduction in the ungated MMCTAC images may indicate the utility of this algorithm should be investigated outside of the standard DDG clinical protocol for high motion patients which gates bed positions with R > 15. Following the DDG protocols allowed for a valid comparison across the reconstruction types however CT misregistration is not limited to high respiratory motion patients [18]. A future study could extend this to include patients just below this threshold to discern the benefits of MMCTAC on ’medium’ or ’low’ respiratory motion patients [19]. Future work may also consider including a broader artefact scoring scale for clinician’s to better reflect more marginal motion correction gains.

The statistically significant increase in lesion SUVmax we observed across all three reconstruction types (*p* < 0.05) aligns with the reader’s rank-order preferences, in line with the assumption that higher SUVs correlate with improved diagnostic confidence and lesion detectability [20].

The pairwise comparisons between the reconstruction types demonstrate that the Q.Static MMCTAC SUVmax was significantly better than both ungated reconstruction methods, with the combined application of DDG and MMCT producing the highest SUVmax. As expected, Q.Static reconstructions exhibited image quality comparable to a half-count 3-minute static reconstruction (*p* = 0.32). However, clinical scoring showed that Q.Static reconstructions achieved lesion detectability and diagnostic confidence comparable to the full-count 6-minute reconstruction despite using only 50% of the acquired counts, due to Q.Static’s motion correcting gating.

Unlike previous work, our study lacked an external gold standard for phase matching so we instead relied on SUVmax and reader preference to assess the performance with FDG data. While this reflects the clinical acquisitions being directly compared since 4DCT is rarely acquired outside radiotherapy planning, it does leave a residual uncertainty about the deformation field produced by the MMCT algorithm. Future multicentre work could include a small 4DCT subset or digital anthropomorphic phantoms.

Including larger lesions within this study would have increased the number of lesions available for analysis and scoring however the number of patients with lesions and high motion remained the same. This study seeks to investigate the efficacy of MMCTAC to improve lesion detectability and diagnostic confidence, for which only borderline smaller lesions are impacted. For a larger study, investigating larger lesions could be further evaluated to identify a lesion diameter threshold to assess the impact of this reconstruction pipeline, building on previous work [14].

Another barrier to drawing more robust conclusions from this study stems from the number of tied pairs in the clinician scoring. The statistical testing therefore lost power and a wider cohort or more discriminatory means of qualitative clinician assessment is required for further investigation into these results. This is reflected in the results of the Wilcoxon signed-rank testing between normAC and MMCTAC for Static 6 min and Q.Static reconstructions to consistently yield insignificant results ( *p>* 0.05). Discussions with the clinician to determine the discriminatory nature of the preference indicated in rank but not in the three scored metrics highlighted that image noise was often the determining factor for the Image Quality score. This resulted in similar scores across attenuation correction methods for the same reconstruction and inability to yield a *p* value from the Wilcoxon signed-rank test. The Likert scoring can obscure the more nuanced benefits indicated by clinician rank. As MMCT is deployed with larger datasets and different tracers are considered, further ways to illuminate the more subtle differences in clinician preference need to be explored to better appreciate the radiologists’ interpretation of the corrected images. These benefits would also be better seen in a multi-reader study given the qualitative nature of the clinician scoring. More than one set of clinician’ scores would provide more robust evidence in favour of the MMCT algorithm if multiple radiologists agreed on its positive motion correction ability.

## Conclusion

MMCT offers a pragmatic, device-free solution for FDG PET/CT phase-matching and respiratory motion correction without complicating established workflows. This has been demonstrated in 16*%* of our sequential cohort, in high motion patients with small liver and/or lung lesions. Overall, the motion-matched CTAC image scores demonstrated the algorithm’s efficacy at reducing the motion artefacts present in FDG PET/CT images, improving SUVmax of lesions in the thorax and upper abdomen. In FDG studies it boosts lesion SUV and demonstrates clinical preference; however, confirmation in larger, multi-reader, ground-truth-referenced cohorts would allow for the degree of improved attenuation-correction alignment to be better quantified.

## Data Availability

The analysed data may be made available by the corresponding author upon reasonable request.

## Abbreviations

AC: Attenuation correction
CT: Computed tomography
CTAC: CT attenuation correction
DDG: Data-driven gating
FDG: Fluorodeoxyglucose
FMISO: Fluoromisonidazole
MMCT: Motion-Matched CT
MMCTAC: Motion-Matched CT Attenuation Correction
normAC: Normal AC i.e. Standard clinical helical CT-based attenuation correction
PET: Positron emission tomography
Q.Clear: GE HealthCare’s BSREM Iterative Reconstruction Algorithm
Q.Static: GE HealthCare’s commercial implementation of Data-Driven Gating
ROI: Region of interest
SUV: Standardized uptake value
SUVmax: Maximum standardized uptake value
